# An alternative route of bacterial infection associated with a novel resistance locus in the *Daphnia–Pasteuria* host–parasite system

**DOI:** 10.1038/s41437-020-0332-x

**Published:** 2020-06-19

**Authors:** Gilberto Bento, Peter D. Fields, David Duneau, Dieter Ebert

**Affiliations:** 1 Department of Environmental Sciences, Zoology, University of Basel, Vesalgasse 1, 4051 Basel, Switzerland; 2 Université Toulouse 3 Paul Sabatier, CNRS, UMR5174, EDB (Laboratoire Évolution & Diversité Biologique), Toulouse, France

**Keywords:** Evolutionary biology, Evolutionary genetics

## Abstract

To understand the mechanisms of antagonistic coevolution, it is crucial to identify the genetics of parasite resistance. In the *Daphnia magna*–*Pasteuria ramosa* host–parasite system, the most important step of the infection process is the one in which *P. ramosa* spores attach to the host’s foregut. A matching-allele model (MAM) describes the host–parasite genetic interactions underlying attachment success. Here we describe a new *P. ramosa* genotype, P15, which, unlike previously studied genotypes, attaches to the host’s hindgut, not to its foregut. Host resistance to P15 attachment shows great diversity across natural populations. In contrast to *P. ramosa* genotypes that use foregut attachment, P15 shows some quantitative variation in attachment success and does not always lead to successful infections, suggesting that hindgut attachment represents a less-efficient infection mechanism than foregut attachment. Using a Quantitative Trait Locus (QTL) approach, we detect two significant QTLs in the host genome: one that co-localizes with the previously described *D. magna* PR locus of resistance to foregut attachment, and a second, major QTL located in an unlinked genomic region. We find no evidence of epistasis. Fine mapping reveals a genomic region, the D locus, of ~13 kb. The discovery of a second *P. ramosa* attachment site and of a novel host-resistance locus increases the complexity of this system, with implications for both for the coevolutionary dynamics (e.g., Red Queen and the role of recombination), and for the evolution and epidemiology of the infection process.

## Introduction

Host–parasite interactions are thought to be one of the main drivers of organismic evolution, promoting both diversification and genetic diversity (Schmid-Hempel 2011). The theory is that hosts evolve to minimize fitness costs associated with parasitism, whereas parasites evolve to maximize fitness while exploiting the host and avoiding its defense mechanisms. Different evolutionary models have been proposed to underlay such host–parasite coevolution, including negative frequency-dependent selection (NFDS), selective sweeps, and heterozygote advantage (Ebert 2008; Wilfert and Jiggins 2013; Papkou et al. 2016). However, as few naturally coevolving host–parasite systems have been sufficiently explored, the genetic mechanisms of these systems are largely unknown (Tiffin and Moeller 2006; van Oosterhout 2009; Ejsmond and Radwan 2015, Wilfert and Jiggins 2013, Bento et al. 2017).

Parasite infection is a complex process that typically requires multiple steps for successful completion of the parasite’s life cycle, i.e., infection, within-host reproduction, and transmission (Hall et al. 2017). With each step, the parasite must overcome the host’s defense mechanisms to continue its life cycle, whereas the host’s degree of success at each step results in different fitness and evolutionary outcomes. A single infection-blocking step can render the host totally resistant, even if the other steps would allow the parasite to proceed. Identifying and studying specific infection steps can, thus, illuminate the complex attack and defense portfolios of specific host–parasite coevolutionary interactions (Hall et al. 2017, Lievens et al. 2018).

**Fig. 1:**
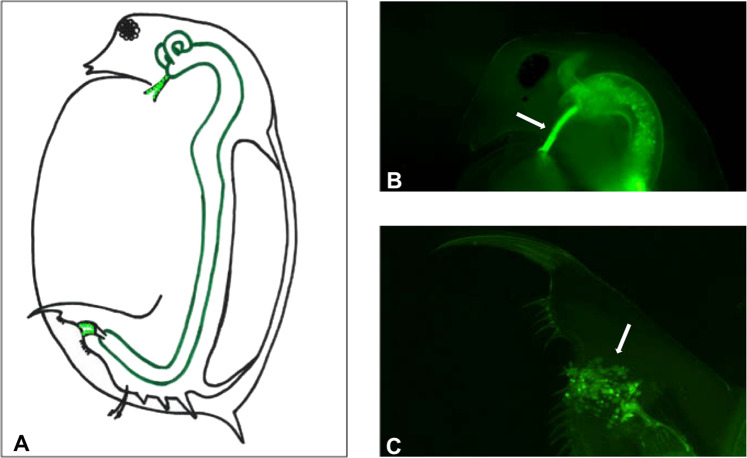
Attachment of *Pasteuria ramosa* to *Daphnia magna* host. **a** *D. magna* host with attachment sites for *P. ramosa* spores (green dots) attached to the foregut and hindgut, but not to the gut midsection (dark-green lining). *P. ramosa* spores marked with a green fluorescent dye (and indicated by white arrows) may attach to the foregut (**b**) or to the hindgut (**c**) of the *D. magna* host.

While we rarely have the necessary information to understand the role each step plays in the infection process and in the coevolution of host and parasite, this information does exist for the *Daphnia magna*–*Pasteuria ramosa* host–parasite system, where recent studies have identified the attachment step as crucial (Duneau et al. 2011, Luijckx et al. 2013; Metzger et al. 2016; Bento et al. 2017, reviewed in Ebert et al. 2016) (Fig. 1). Early in the infection process, *P. ramosa* spores are activated, shedding their protective exosporium. Next, the activated spores attach to the host’s foregut (Fig. 1a,b) and penetrate into its body cavity where they reproduce and eventually kill the host. When foregut attachment fails, the host is resistant (Duneau et al. 2011). Variation in attachment success explains most variance in host–parasite interaction (Ebert et al. 2016). Studies have shown that the host–parasite interaction at the attachment step follows a matching-allele model (MAM), whereby infection of resistance can only occur when specific host and parasite alleles meet. MAM is one of the mechanisms that have been proposed to avoid the occurrence of super-genotypes, i.e., parasite genotype, which are universally infectious, or host genotypes, which are universally resistant (Lively and Dybdahl 2000; Hamilton 1980; Clarke 1976). Thus, in following a MAM, the *Daphnia*–*Pasteuria* host–parasite system fulfills a key assumption of coevolution by NFDS (Decaestecker et al. 2007; Duneau et al. 2011; Luijckx et al. 2012, Metzger et al. 2016; Bento et al. 2017).

The genetics of *D. magna*’s resistance to *Pasteuria* spore attachment has received the most attention in the context of parasite genotypes C1 and C19 where one genomic region, the *Pasteuria* resistance (PR) locus, has been found to underlie variance in *D. magna* resistance to these *P. ramosa* genotypes. In addition, the natural variation at the PR locus has been found to match the predicted MAM (Bento et al. 2017). In this study, we report a newly isolated *P. ramosa* genotype, P15, that uses a different entry point into the host—the hindgut. We investigate *D. magna’s* resistance to this *Pasteuria* isolate and discuss our findings on the infection mechanism and on the coevolutionary dynamics of this interaction.

## Methods

### Hosts and parasites

Two different *D. magna* genetic panels have been used in this study.

Diversity panel: The *Daphnia magna* Diversity Panel is a growing collection of *D. magna* genotypes collected across the entire species range (corresponding approximately to the entire Holarctic) with one genotype per population. All clones are kept clonally in laboratory standard medium (ADaM, [Klüttgen et al. 1994] modified by using only 5% of the recommended selenium dioxide concentration) on a diet of green algae (*Scenedesmus* sp.) at a temperature of 20 °C and a light: dark cycle of 16:8 (Luijckx et al. 2011). This panel has been used in previous studies (Roulin et al. 2013; Yampolsky et al. 2013; Seefeldt and Ebert 2019), but continues to expand with new *D. magna* clones. In this study, we used females from 174 clones. The diversity panel was used in this study to investigate the natural diversity of *D. magna* resistance to attachment and infection by *P. ramosa* P15 genotype.

Recombinant panel: The F2-recombinant QTL panel used in this study was developed by Routtu et al. (2010) and was kept as clonal lines in the laboratory. In brief, this panel originated from the crossing of two divergent *D. magna* parent clones, one from a Finnish rock pool population (Xinb3) and the other from a pond near Munich, Germany (Iinb1). One F1 offspring was cloned and selfed to produce the F2-recombinant clones. These F2-recombinant clones were typed at about 1300 SNP markers to produce a genetic map (Routtu et al. 2014). Our F2-recombinant QTL panel in this study consisted of two subpanels: the core recombinant panel, a set of randomly chosen F2-recombinant clones, and the extended recombinant panel, a set of F2-recombinant clones selected for their susceptibility to the C19 *Pasteuria ramosa* genotype (among randomly chosen clones, only 25% are susceptible to this parasite genotype) (see Routtu et al. 2014 for more details). Here we used 208 core panel and 169 extended panel F2-recombinant clones to map and identify the genetic basis of *D. magna* resistance to *P. ramosa* P15.

Three *P. ramosa* parasite genotypes were used in this study: C1 and C19 clones were derived from natural isolates of *P. ramosa* from Moscow, Russia, and Gaarzerfeld, North Germany (see Luijckx et al. 2011), respectively, and have only been observed to attach to the foregut of the host. P15 was isolated from a sample collected in Heverlee, Belgium. It was not cloned, but was passaged multiple times in a susceptible *D. magna* host clone. For simplicity, we call it here the P15 genotype, but we cannot exclude the possibility that this isolate may contain some within isolate genetic variance. P15 typically attaches to the hindgut of the host, although we have observed a few cases where it attaches to the foregut.

### Attachment test

The attachment test is a fluorescence-based assay that tests the ability of *Pasteuria* spores to attach to the fore- or hindgut of the host (Duneau et al. 2011) (Fig. 1b, c). Attachment indicates the parasite’s ability to infect the host, while absence of attachment indicates resistant hosts (Duneau et al. 2011). Attachment is necessary for infection, but hosts may still be resistant if they can either prevent penetration (Duneau and Ebert 2012) or efficiently clear the infection (Hall et al. 2012) in later steps.

For the attachment tests, we exposed young host individuals to fluorescently labeled *Pasteuria* spores. After 30–60 min, the transparent hosts were observed under a fluorescent light to check if spores attached to their esophagus (foregut) or hindgut (Fig. 1b, c). More details of this method are provided in Duneau et al. (2011). Because the scoring of hindgut attachment phenotype is more difficult than the scoring of foregut attachment, there may also be an increased rate of false positives and negatives. We increased the number of replicates of the hindgut attachment (median = 12, max = 39, and min = 6) assays relative to foregut attachment (median = 9, max = 27, and min = 6) in order to increase our power to discern attachment success. All attachment tests were performed without the observer knowing the genotype of the animal being tested. All *D. magna* genotypes of the two genetic panels mentioned here were tested for their resistance to attachment by *P. ramosa* P15. For the purpose of these experiments described here, attachment was defined as a binary trait and clones classified as either resistant (no attachment) or susceptible (attachment).

### Infection test

To test whether the infection assay protocol developed for foregut-attaching *P. ramosa* genotypes (Ben-Ami et al. 2010) would also result in successful P15 infections, we selected 40 clones from the QTL panel. Twenty one of these clones showed no P15 hindgut attachment in all tested replicates (P15_hindgut_R), and 19 clones showed P15 hindgut attachment in most or all replicates (P15_hindgut_S). The hindgut infection assay used here followed a modified version of previously published protocols for *Pasteuria* infections (Ben-Ami et al. 2010). The protocol modification consisted of individually cultured juvenile *D. magna* being exposed daily to a parasite spore dose high enough to infect susceptible hosts, i.e., those *D. magna* host genotypes prone to foregut-attaching *Pasteuria* with 90–100% efficiency (Ben-Ami et al. 2010). This was done to avoid the removal of attached bacterial spore during molting (Duneau and Ebert 2012). In short, hosts were exposed to 50,000 parasite spores per day over 3 days, resulting in a total of 150,000 spores per animal. Exposed *D. magna* was fed daily with *Scenedesmus sp*. and kept individually in 80 mL medium (in 100 mL jars) under the same conditions as those in which it was raised (described above). After 40 days, we assessed the number of infected animals. Animals that died during the course of the experiment were not included in the analysis. The final dataset includes, on average, 19.03 replicates per clone. For the purpose of the experiments described in this paper, only the *D. magna* genotypes of the diversity panel were tested for their resistance to *P. ramosa* P15 infection.

### Genetic mapping

Replicates (mean = 6 replicates, range: 3–15) of each of the 379 F2-recombinant clones of the F2 QTL panel were tested for P15 spore attachment (see “Attachment test” section). See Routtu et al. (2010) for a description of the structure and construction of the SNP array linkage map used. The QTL analysis was performed with R package *R/qtl* version 1.29–2 (Broman et al. 2003), following the same analysis as described in Routtu and Ebert (2015) and Krebs et al. (2017) and using the same SNP map and QTL panel. In short, Haley–Knott regression (Knott, Haley 1992) was used for robustness and speed of analysis. The assumptions of the model were investigated and confirmed. To find epistatic interactions, we run scantwo (for multidimensional scans with a multiple-QTL model). Finally, using option fitqtl, a defined multiple-QTL model was analyzed (Broman et al. 2003). A genome-wide significance level was established using 10,000 permutation tests with significant (α = 0.05) LOD scores of 3.78. Analysis of variance was used to estimate the proportion of the total variance explained by the fitted models.

### Fine mapping

To fine-map the D locus, clones generated in the Recombinant Panel were scored independently for attachment by *P. ramosa* P15 spores with two scoring methods: using a binary definition of attachment (resistant or susceptible) as described above and using a continuous definition of attachment where strength of attachment was classified by the observer in a scale from 1 (no attachment) to 10 (strongest attachment). For further details see S1 Methods.

We validated the continuous definition of attachment by testing the correlation between the mean of the binary scoring and the median of the quantitative attachment scoring, using the 347 clones of the recombinant panel, which were tested with both methods. The results show that the binary and continuous methods are consistent with each other, and that the correlation is strong (Pearson *r* = 0.73, *n* = 347, *p* < 0.001).

For the fine mapping, we selected only those F2 clones that were scored either fully resistant or fully susceptible with both scoring methods before proceeding with the breakpoint mapping. For further details see S1 Methods.

### Sequencing, assembly, and annotation of D locus

The methods for sequencing, assembling, and annotating the Xinb3 and Iinb1 D-locus haplotypes were identical to and are described in detail in Bento et al. (2017). In short, because the region around the QTL where D locus is located was poorly assembled in version 2.4 of the *D. magna* draft genome (http://wfleabase.org/), we undertook a number of additional sequencing and assembly methods in order to better resolve the focal region. For Xinb3, we generated high- coverage (~60×) PacBio sequencing in order to perform de novo genome assembly. For Iinb1, we took a hybrid Illumina short-read/PacBio long-read approach, generating ~80 × 125 bp PE Illumina coverage and ~15× PacBio long-read coverage. We used the *D. magna* Xinb3 and Iinb1 haplotype sequences obtained to blast search homologies within and between haplotypes and other genomic regions.

In order to understand how expression of individual genes localized to the focal genome regions and to other parts of the genome differed between the Xinb3 and Iinb1 clones, we conducted a de novo transcriptome assembly of the dataset described in Orsini et al. (2016). Finally, we constructed a de novo annotation of each of the transcripts mapping to the D locus by performing blastx (nucleotide-to-protein) searches in the NCBI database. For details on the methods for DNA extraction, genomic sequencing and assembly, and de novo transcriptome assembly mapping annotation see S2 Methods.

## Results

### *P. ramosa* P15 attaches to *D. magna* hindgut and causes infection

Of the 174 genotypes from the diversity panel that we tested for *P. ramosa* P15 attachment, most cases of positive spore attachment occurred in the hindgut of the host (Fig. 1c). Out of 2241 *D. magna* host individuals tested, 1197 (53%) showed P15 attachment in the hindgut, while 163 (7%) animals showed attachment to the host’s foregut (Table S1). In contrast, testing the same 174 *D. magna* genotypes for attachment of *P. ramosa* clones C1 and C19, we observed only polymorphism for foregut attachment (Table S1), with no cases of hindgut attachment.

**Fig. 2:**
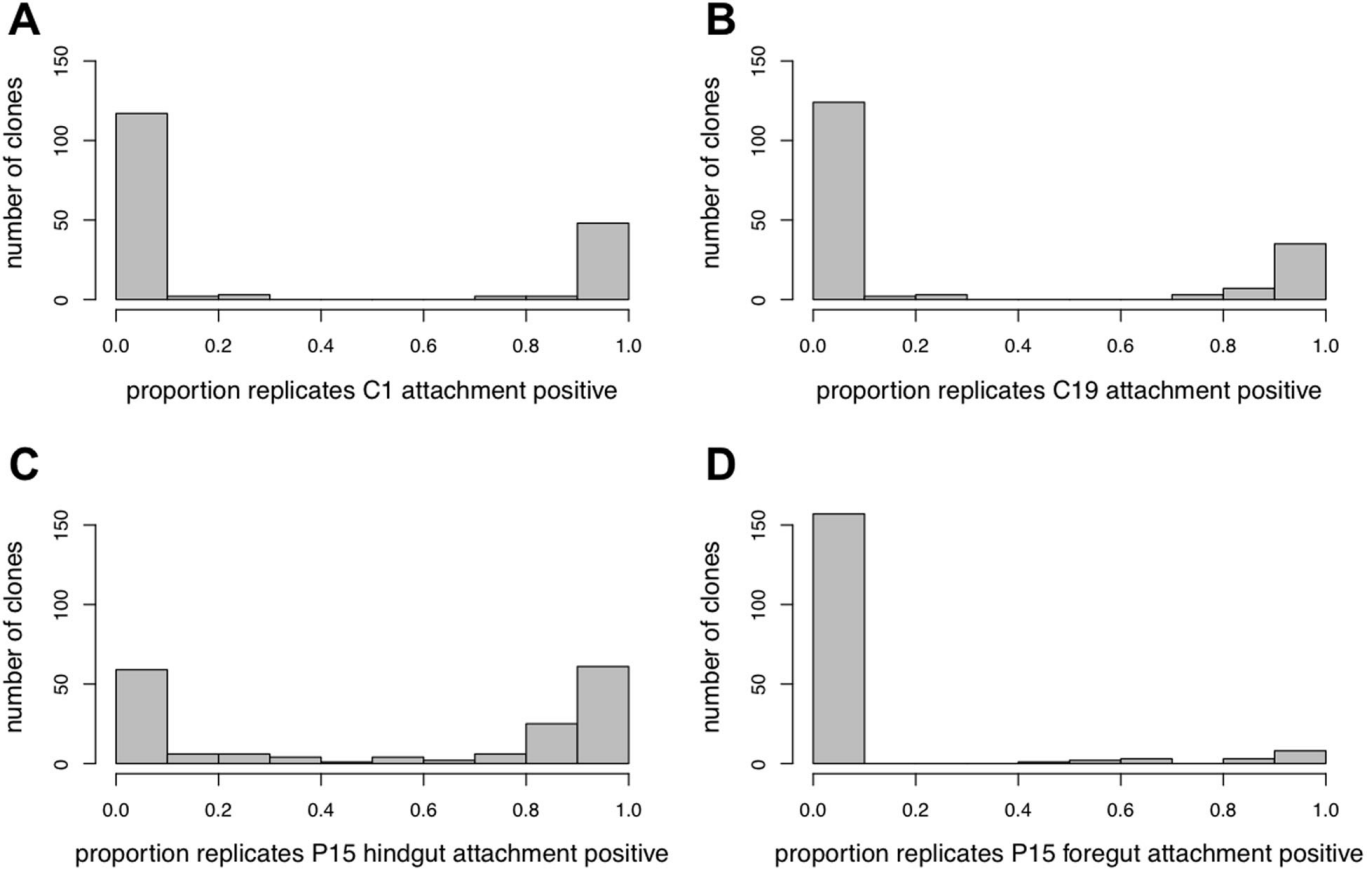
Variation of *Pasteuria ramosa* attachment success across 174 genotypes of the *Daphnia magna* diversity panel. Graphs show the proportion of positive attachment tests across replicates within genotype on the x axis and the number of *D. magna* genotypes (=clones) for each category on the y axis. The four graphs show *P. ramosa* C1 attachment to the host foregut (**a**), C19 to foregut (**b**), P15 to hindgut (**c**), and P15 to foregut (**d**).

A hallmark of *Pasteuria* foregut attachment is that host genotypes (clones) either allow attachment or not, with very little within-clone phenotypic variance, i.e., inconsistent attachment test results between replicates from the same host genotype are rare (Duneau et al. 2011). However, P15 attachment to the hindgut appeared more variable: out of 174 *D. magna* genotypes tested, 59 (34%) were attachment-negative in all replicates, 47 (27%) were attachment-positive in all replicates, and 68 genotypes (39%) show variance among replicates of the same host genotype (with a minimum of six and an average of 12.8 individuals tested per host genotype) (Fig. 2). In testing the same 174 clones for C1 and C19 attachment, we found no within-clone variance for C1 and little variance for C19 attachment (<10% of host clones, Fig. 2). Thus, we observed more quantitative variance for *P. ramosa* P15 hindgut attachment than for C1 and C19 foregut attachment. Nevertheless, the distribution of P15 attachment frequencies showed a pronounced bimodality (Fig. 2).

*P. ramosa* P15 attachment to the foregut was observed in about 7% of the Diversity panel host individuals. P15 foregut attachment was also variable within clones, but clearly bimodal (Fig. 2). Of those 17 *D. magna* genotypes where foregut attachment occurred in at least some replicates, eight showed attachment in all replicates (Fig. 2). Twelve of the 17 were resistant to P15 hindgut attachment, while the remaining five were able to attach to both the foregut and the hindgut (Table S1).

**Fig. 3:**
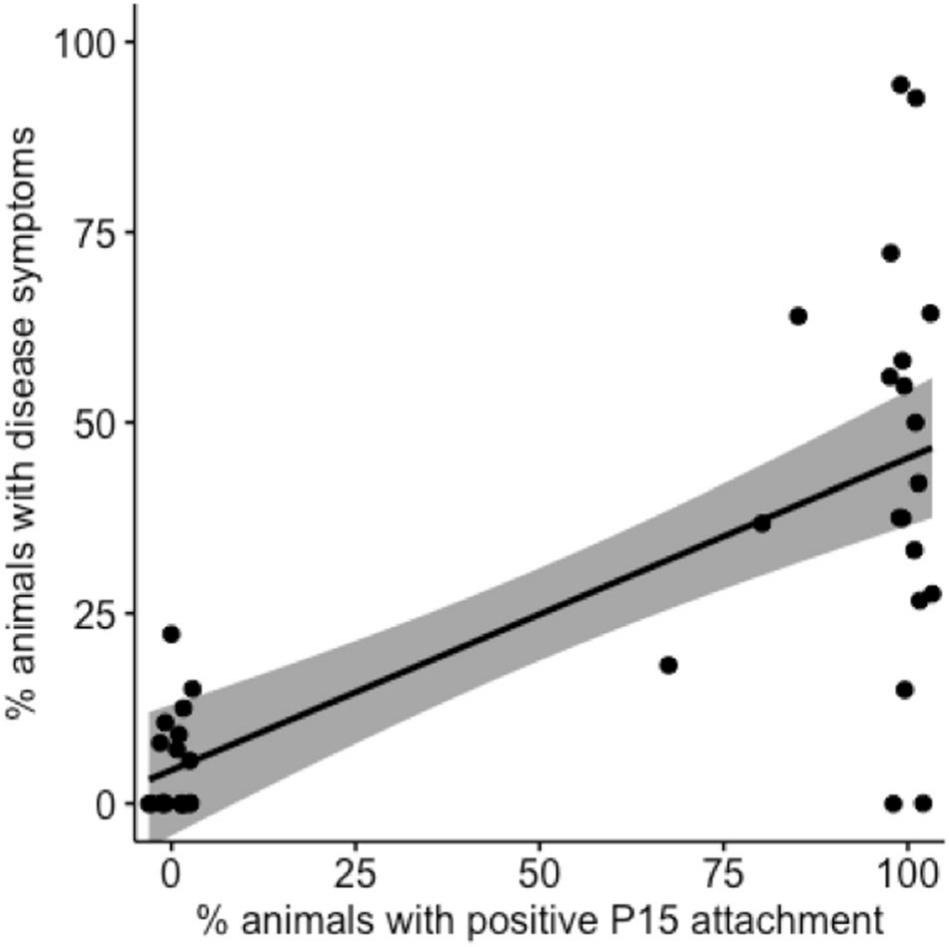
Correlation of Pasteuria ramosa *P15 attachment to the hindgut and the percentage of exposed animals that became infected*. P15 hindgut attachment and infection success in 40 *Daphnia magna* genotypes (Spearman’s rho = 0.73, *p* < 0.001, *n* = 40).

Attachment of *P. ramosa* C1 and C19 genotype spores to *D. magna* foregut was shown to be necessary for successful infections; indeed, infection nearly always follows C1 and C19 spore attachment (Duneau et al. 2011). We tested whether this held true for *P. ramosa* P15 attachment to the *D. magna* hindgut (Table S2) by using P15 attachment-positive and P15 attachment-negative clones from a standing QTL panel (Routtu et al. 2014) and exposing 40 host clones to infectious P15 spores. The P15 attachment-negative clones remained largely uninfected (median infection rate: 0, range: 0–22.2%), whereas most P15 attachment-positive clones showed infection (median infection rate: 42.1%, range: 0–94.4%) (Fig. 3). We found a strong correlation between the attachment results and successful infection (Spearman’s rho = 0.729, *P* < 0.001, *n* = 40).

### QTL analysis and Mendelian segregation

Our investigation into the genetic architecture of *D. magna* resistance to P15 hindgut attachment using the F2-recombinant panel suggested that P15 attachment is dominant, as the F1 clone showed attachment. The parent clone Xinb3 has perfect attachment to P15, whereas parent clone Iinb1 has no attachment. Of the 377 F2 clones of the QTL panel, 51% of the genotypes showed within-clone variance, whereas 37% showed attachment in all replicates, and 12% showed no P15 spore attachment (Table S3).

**Fig. 4:**
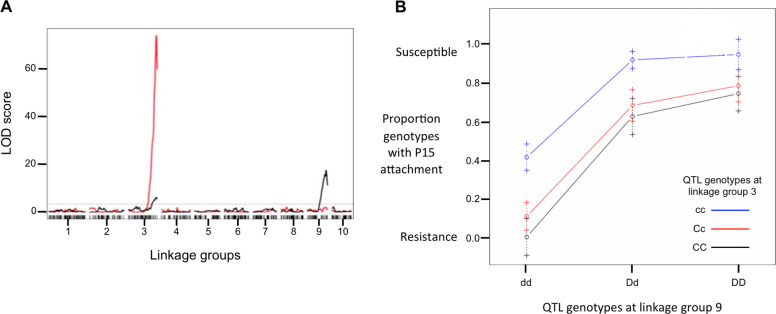
Quantitative Trait Locus analysis of P. ramosa attachment to the host *Daphnia magna*. **a** LOD scores for Pasteruia attachment plotted against the entire genome of Daphnia magna. *P. ramosa* C19 attachment to the *Daphnia magna* foregut shown in red and *P. ramosa* P15 attachment to the hindgut shown in black. The dashed line represents the significance threshold at *P* = 0.05. **b** Effect plot for P15 attachment of host genotypes at QTL detected in lg3 and lg9. The German parent clone (Iinb3) of the QTL panel has genotype CC at lg3 and dd at lg9, whereas the Finnish parent clone (Xinb3) has genotype cc at lg3 and DD at lg9. Variation shown along the x axis corresponds to the D locus, while the three colored lines represent variation at the C locus.

The QTL analysis revealed two large-effect loci associated with P15 attachment in *D. magna* that together accounted for 42.1% of the total observed variance within the mapping population (Fig. 4a) (Table 1). One locus explained 11.8% of the variance and co-localizes with the previously described foregut attachment QTL on linkage group (lg) 3 (Routtu and Ebert 2015). This lg has been shown to harbor the PR locus for genetic variance in resistance to foregut attachment by C1 and C19 (Bento et al. 2017). The second QTL was found on lg9 and explained 30.3% of the total variance within the mapping population (Table 1). The effect plot (Fig. 4b) shows that the allele from the susceptible Finnish parent clone (Xinb3) at the second QTL is dominant for positive attachment and the allele from the resistant German parent clone (Iinb1) is recessive for no attachment. In maintaining our previous nomenclature of *P. ramosa* resistance loci (Metzger et al. 2016; Bento et al. 2017), we named this resistance locus the D locus. The interaction between the two QTLs on lg3 and lg9 was not significant (Table 1). Thus, there is no evidence for epistasis, and the joint effect of the two QTLs is therefore considered to be additive (Fig. 4b).

**Table 1:** Quantitative Trait Loci as detected by analysis of *Pasteuria ramosa* P15 attachment to *Daphnia magna*.

	df	SS	LOD	%var	*F* value	*P* value (Chi^2^)	*P* value (F)
Lg 3 SNP: scaffold00288_965	6	374.55	14.34	12.29	11.84	<0.001	5.05e^–12^
Lg 9 SNP: scaffold02269_730	6	957.68	32.21	31.42	30.28	<0.001	<2e^–16^
Lg 3 × lg 9	4	16.07	0.6771	0.53	0.76	0.54	0.551

As seen in the natural host isolates (Fig. 2), there was within-clone phenotypic variance among the F2 clones for P15 attachment (Table S3). However, overall attachment frequencies for P15 and C19 showed clear bimodal distributions. By categorizing attachment-positive clones (P15_hindgut_S) as those in which more than 50% of the replicates showed attachment, and attachment-negative clones (P15_hindgut_R) as all other clones, we were able to test the F2 clone data for Mendelian segregation. The F2 core panel (*n* = 208) showed Mendelian segregation for resistance to C19, with 73.6% of the F2 clones being C19-negative and 26.4% being C19-positive (Table 2), consistent with a 3:1 Mendelian segregation ratio (*χ*^2^ = 0.865, *P* = 0.715). C19 attachment-positive clones have the CC or Cc genotype at the C locus and C19 attachment-negative clones are homozygotes for the recessive c allele (Metzger et al. 2016; Bento et al. 2017).

**Table 2:** *D. magna* F2-recombinant panel results for the core and the extended panel and for the genetic model for *P. ramosa* C19 and P15 genotypes.

Core panel (208 F2 clones)		
	P15 attachment negative	P15 attachment positive
	D-locus genotype: dd	D-locus genotype: DD or Dd
C19 attachment negative -C-locus genotype: CC or Cc	C19_P15 resistotype: RR genotype: CCdd or Ccdd 31.25% (*n* = 65)	C19_P15 resistotype: RS genotype: CCDD, CcDD, CCDd, or CcDd 42.3% (*n* = 88)
C19 attachment positive C-locus genotype: cc	C19_P15 resistotype: SR genotype: ccdd 4.81% (*n* = 10)	C19_P15 resistotype: SS genotype: ccDD or ccDd 21.6% (*n* = 45)
Extended panel (169 F2 clones)		
	P15 attachment negative	P15 attachment positive
	D-locus genotype: dd	D-locus genotype: DD or Dd
C19 attachment positive C-locus genotype: cc	C19_P15 resistotype = SR genotype: ccdd 22.5% (*n* = 38)	C19_P15 resistotype = SS genotype: ccDD or ccDd 77.5% (*n* = 131)

The assessed resistotype (C19 P15), the inferred genotypes for the C locus (alleles: C and c) and the D locus (alleles D and d), and the percentage of host F2 clones for each resistotype are given. Capital letters of the inferred genotypes indicate dominant alleles. The data underlying this table are presented in Table S3.

The 169 F2 clones of the extended QTL panel, which includes only C19 attachment-positive clones (i.e., homozygote cc), showed Mendelian segregation for P15 attachment resistance, with 77.5% being P15-positive and 22.5% being P15-negative (Table 2, extended panel). This coincides with a 3:1 Mendelian segregation ratio, with positive attachment (susceptibility) being dominant (*χ*^2^ = 0.226, *P* = 0.682). Thus, we can infer that P15 attachment-positive clones have DD or Dd genotypes at the newly defined D locus, and P15 attachment-negative clones have the recessive dd genotype at the D locus. However, in the core QTL panel, the proportion of P15 attachment-positive clones is only 63.9% (Table 2), representing a significant departure from the Mendelian segregation ratio (*χ*^2^ = 5.086, *P* < 0.001). If we consider only F2 clones that are C19 attachment-negative (genotypes CC or Cc), this number declines further to 42.5% of clones being P15 attachment-positive (Table 2). Thus, the dominant resistance allele at the C locus in lg 3 increases the likelihood that the host genotype will show resistance to P15 attachment, a result consistent with the effect plot of the QTL analysis (Fig. 4b).

### Fine mapping and genomic characterization of the D locus

Following the scoring of the attachment of *P. ramosa* P15 spores to the hindgut of the *D. magna* F2-recombinant clones with the binary and the qualitative method (Table S4), we selected only those *D. magna* clones that were fully susceptible or fully resistant with both scoring methods and were left with 112 genotypes (Table S5). The breakpoint mapping using these genotypes determined the boundaries of the large-effect QTL on lg9 where the D locus is located. The genomic region, which was identified, has ~13 kb between SNP markers scaffold01547_75 and scaffold02269_730 (Table 3) (Table S5). We used PacBio long reads to sequence and assemble de novo the D-locus haplotype in the parental clones (Xinb3 and Iinb1) of the F2 QTL panel, designating these two haplotypes as xD locus (Xinb3 parent clone) and iD locus (Iinb1 parent clone), respectively.

**Table 3:** Single-nucleotide polymorphism marker sequences.

Marker ID, comment	Sequence
scaffold00288_965, best hit on lg3	TTGTTAAAGTCCATTGTaAGTGTTTAAGTAGCAAA
scaffold02269_730, best hit on lg9, and flanking SNP on lg9	CATTTCGTCCTGAAaATATGTCACATTGTGTT
scaffold01547_75, flanking SNP on lg9	AaTAAAATCTTAAAAACACaAAAGAAAATTCGTCCAA

Lowercase letters indicate SNPs. Flanking SNPs mark the markers closed to the D locus on lg9.

By producing a de novo *D. magna* transcriptome and using reciprocal blasts between the *D. magna* transcriptome and the newly assembled D-locus haplotypes to map and annotate expressed genes in this region, we found six expressed genes in the xD-locus haplotype (Table 4).

**Table 4:** *Daphnia magna* expressed transcripts and predicted genes mapping to the D locus.

Predicted gene	Transcript	Length (bp)	Up/downregulated in Xinb3 parental clone
*Dm* UP LOC116927272 ncRNA	TRINITY_DN1103_c0_g1_i1	871	NS
*Dm*-probable ATP-dependent RNA helicase spindle E	TRINITY_DN20188_c3_g1_i1	4337	Upregulated
	TRINITY_DN20188_c3_g1_i2	1408	Downregulated
	TRINITY_DN358_c0_g1_i1	340	NS
*Dm* calcyclic binding protein-like mRNA	TRINITY_DN20044_c2_g1_i6	2368	NS
*Daphnia magna* protein SMG7-like	TRINITY_DN10965_c0_g1_i1	1392	NS
*Dm* UP LOC116927249 ncRNA	TRINITY_DN6503_c0_g1_i1	628	NS
*Dm* MIT domain protein 1-like mRNA	TRINITY_DN10965_c0_g1_i1	1392	NS

*NS* nonsignificant.

In comparing the RNAseq database produced by Orsini et al. (2016) (based on the whole-body gene expression of the Xinb3 and Iinb1 parent clones of our QTL panel) with the gene expression at the D locus, we found that one transcript significantly upregulated in the xD locus, and one transcript that was significantly downregulated. The two transcripts, which were differentially regulated, correspond to two isoforms of a *D. magna*-probable ATP-dependent RNA helicase spindle E. Transcript TRINITY_DN20188_c3_g1_i1 (4337 bp long), which is upregulated in the Xinb3 P15-susceptible host clone, and the transcript TRINITY_DN20188_c3_g1_i2 (1408 bp long), which is downregulated in the Xinb3 P15-susceptible clone. Neither of them has, to our knowledge, been associated with resistance against a pathogen.

## Discussion

Bacterial attachment to the host’s foregut has been shown to be a crucial step in the infection process of *D. magna* by *P. ramosa* parasites. Failure in this step blocks infection altogether, whereas success nearly guarantees infection (Duneau et al. 2011). Here we describe an alternate location of parasite entry into the *D. magna* host—attachment to the host’s hindgut. This route has not been described before and is used by a different genotype (P15) of *P. ramosa*. Interestingly, P15 is also able to use the foregut as an attachment site in some cases.

The discovery of a second entry point for *P. ramosa* into its host adds complexity to our understanding of the mechanisms and evolution underlying host resistance to *Pasteuria* infections. Our previous picture relied on the assumption of a linear stepwise infection process: (1) host–parasite encounter, (2) activation of the parasite endospore, (3) attachment to the host cuticle, (4) penetration of the host, (5) early and (6) late within-host growth phase, and (7) host death (Ebert et al. 2016, Hall et al. 2017). Our new finding suggests that this process bifurcates after the activation step (step 2), with parasites being able to attach (step 3) and penetrate (step 4) at one of two entry points, or, in some cases, at both points. Following host penetration, parasites that enter through both entry points start proliferating within the host’s body cavity (steps 5 and 6), leading finally to the death of the host (step 7). Alternate host entry points are known from other host–parasite systems. For example, anthrax infections in humans are caused by *Bacillus anthracis*, which has multiple ways to enter a host, including lung entry after the spores are inhaled, cutaneous anthrax after skin contact with spores, and gastrointestinal anthrax after food-borne spore uptake (www.cdc.gov/anthrax/basics/how-people-are-infected.html). In the *Daphnia* system, the microsporidium *Hamiltosporidium tvaerminnensis* is able to enter the host by transovarial infections and through the ingestion of free spores with the food (Vizoso and Ebert 2005). Unless different infections routed are traded off with each other (Lipsitch et al. 1995), parasites generally benefit from having multiple ways to infect a host, as it increases their chances of infection and may reduce the chances for the host to evolve resistance. With the second entry route for *Pasteuria* that we describe here, neither a trade-off nor a widening of opportunities with different ecological conditions for the two routes of entry are known.

Both the foregut and the hindgut of *D. magna* are ectodermic tissues that are part of the animals’ cuticle and that are molted in regular intervals throughout the host’s life. The remaining section of the gut (the midgut) is of endodermic origin and is not molted. As the hind- and foregut tissue stems from a shared ectodermic origin (Schultz and Kennedy 1976) and possibly a shared genetic basis, the attachment mechanism might be conserved in the two infection routes. The observation that P15 can attach to both the hindgut and the foregut, and in some host genotypes even to both sites, further suggests a similarity of the two attachment sites. However, nothing is known about the cuticle surface of those sites in *Daphnia*. The general course of an infection is also similar at both sites. The typical disease symptoms—castration, gigantism, and a life span of 40–50 days post infection—that are observed in response to infections by foregut-attaching *Pasteuria* genotypes (Clerc et al. 2015; Ben-Ami 2017), are also observed in response to infection by the P15 isolate.

Our study also revealed an important difference between fore- and hindgut attachment: we observed that positive attachment (susceptibility) is recessive when tested with *Pasteuria* C19, but is dominant when tested with *Pasteuria* P15. This is, however, not entirely new for this system, as an earlier study demonstrated that in the A locus of the PR supergene (Bento et al. 2017) (which includes the A, B, and C loci), either resistance or susceptibility can show as dominant depending on the *Pasteuria* isolate used and the genotype at other loci (Luijckx et al. 2013). A further difference observed in P15 hindgut attachment is that attachment success followed a more quantitative, but still strongly bimodal, pattern (Fig. 2). About 40% of host clones isolated from natural populations showed within-clone phenotypic variance in hindgut attachment success for *Pasteuria* P15. For the foregut-attaching *Pasteuria* C1 and C19, this percentage is below 10% (Fig. 2). We also observed that the infection rate among P15 attachment-positive host clones was comparatively low and highly variable (Fig. 3). A few host clones remained uninfected even after being exposed to spore doses that would lead typically to nearly 100% infection success for the two foregut-attaching parasites C1 and C19 (Duneau et al. 2011). Whether the reduced infection rate of P15 attachment-positive clones is caused by less-efficient attachment and host penetration, or by subsequent clearing of infections by the host’s immune system (within-host defense), is not clear. Surprisingly, some clone replicates that were scored as consistently P15-hindgut attachment negative developed a full-blown infection after exposure to the spores. This finding may hint at a third, yet-to-be-discovered, entry point into the host.

The lower consistency in hindgut attachment may stem from differences in the attachment mechanism, with potentially weaker cell-to-cell attachment in the host hindgut. It may also be a consequence of the increased difficulties that infective spores have in reaching the hindgut attachment site. The passage through the host’s entire intestinal tract represents an extra step in the infection process and may add variability in the interaction between parasite and host that reduces the number and quality of parasite spores reaching the attachment site. Indeed, as the outer shell (the protective exosporium) of the spores is removed before it is ingested by the host, the host’s defense and the digestive system may alter the integrity of the activated, and hence unprotected, bacteria. Given these variables, stochastic effects likely play a larger role for *Pasteuria* attachment in the hindgut, although other factors may also contribute to attachment success, including the role of undetected minor-effect QTL.

### Hindgut attachment is genetically determined by two loci

Previous genetic studies of natural variation in resistance to C1 and C19 attachment suggest that *D. magna*’s genetic variance in resistance to these *P. ramosa* clones lies within a model of three linked loci (A, B, and C). These loci are localized in the PR locus, which was mapped on linkage group 3 (lg3) (Routtu and Ebert 2015; Metzger et al. 2016; Bento et al. 2017). For one of these loci, the A locus, a matching allele matrix has been described (Luijckx et al. 2012; Bento et al. 2017). For the P15 clone, the QTL analysis of hindgut attachment revealed one locus that co-localizes within or close to the previously described large-effect QTL on lg3 (Routtu and Ebert 2015), as well as a second locus, the D locus, that was newly discovered in this study. This D locus explains most genetic variance within our mapping population in P15 hindgut attachment. Our analysis suggests that the effect of these two QTLs is additive, although this conclusion might change with a larger sample size, as additivity is concluded from the absence of significant epistatic effects. Strong epistasis has been reported for the interaction between the A-, B-, and C locus (Metzger et al. 2016, Bento et al. 2017).

This PR-locus supergene, responsible for variation in foregut attachment on lg3, shows dramatic structural polymorphism between resistant and susceptible *D. magna* clones, including long sequences that are nonhomologous and apparently nonrecombining (Bento et al. 2017). Defined as a genomic region that hosts a number of genes involved in one phenotype, this supergene serves as a hotspot for adaptive evolution and, due to suppressed recombination, behaves genetically like a single gene (Schwander et al. 2014; Bento et al. 2017). The two PR-loci haplotypes differ by ~55 kb and the nonhomologous regions stretch across more than 70 kb (34% of the xPR locus and 46% of the iPR locus) (Bento et al. 2017).

In contrast, the D locus described here showed no major structural differences, and the two D-loci haplotypes are of similar length: xD locus is 13,059 bp long, and iD locus is 12,751 bp long. Also, in contrast to the PR locus, we could not find any extensive repeat structure in the D locus (Bento et al. 2017).

Taken together, this evidence indicates that the D locus is a very different type of genomic region from the unusual structure and polymorphism observed in PR locus, suggesting a different evolutionary and genetic history for these two *Pasteuria* resistance loci.

### The evolution of host–parasite interactions

Our survey of a Holarctic sample of 174 *D. magna* genotypes indicates that resistance polymorphism for P15 attachment is widespread (found within Europe, Asia, North Africa, and North America) and thus should be considered in the evolution of *Daphnia–Pasteuria* interactions. Having two entry points reduces the strength of selection for resistance at each of the points, as the direct link between entry point and infection is weakened, especially when population infection rates are high and multiple infections are common. A further complication is that one of the QTLs described here explains genetic variance for both C19 foregut resistance and P15 hindgut resistance. Thus, the evolution of resistance at the two entry points is not independent from each other.

Our findings also have implications for the evolution of the parasite. Since attachment to the hindgut appears to be weaker and more subject to stochastic variation (as compared with foregut attachment), foregut-attaching parasites have an advantage over hindgut-attaching parasites. Within-host competition may further amplify this advantage, because it is likely to give the competitive edge to the (on average) earlier-infecting parasite genotype (the one with the higher force of infection). On the other hand, the new *Pasteuria* isolate, P15, does have an advantage in that it can attach to both fore- and hindgut, which increases its chances to encounter a susceptible host. It is expected that selection would favor this ability to use additional entry points, unless it comes with costs. Interestingly, we observed that of the 174 host genotypes tested, more than 50% are attachment positive for P15, while only about 30% were positive to attachment with C1 and C19 (Fig. 2). Thus, P15 may be able to compensate for its lower infectivity with a higher attachment rate.

As for coevolution in the *Daphnia*–*Pasteuria* model, this is one of the few examples where a MAM has been observed in a host–parasite system (Bento et al. 2017). This MAM is observed for the segregation of the A locus and only in a specific genetic background: the B locus must be fixed for the dominant B allele and the C locus for the recessive c allele (Bento et al. 2017; Luijckx et al. 2013). The A locus does not segregate in the F2-recombinant QTL panel, and therefore we do not know if the D locus influences the MAM for the A locus. However, the interaction of the D locus with the C locus, which in turn is linked to the A locus, suggests that this might be possible. The increasing number of resistance loci being uncovered in this study system would lower the power of natural selection to change allele frequencies, while the importance of genetic recombination for the resistance phenotype increases. However, the ability to predict resistance phenotypes and the relative influence of selection and recombination in the natural history of the *Daphnia*–*Pasteuria* system is dependent on local diversity patterns. The diversity panel, which we used in this study, is made of genotypes collected from 174 populations from throughout the Holarctic, which maximizes genetic variance, but does not reflect diversity in a local population.

For the moment, we do not know how widespread parasites using different entry points or their combination are in natural populations. But we believe that the finding of P15 hindgut entry adds at least two important pieces to our understanding of the coevolutionary dynamics of the *Pasteuria–Daphnia* system. First, hosts and parasites are more diverse than previously known, therefore providing more opportunities for selection to act on. Second, with the finding of a resistance locus distant from the PR supergene, recombination becomes more important, as it creates further variability in host resistance. Even though this study adds further understanding of the interaction between *D. magna* and its parasite *P. ramosa*, the current picture still misses a lot of the natural variation present in hosts and parasites. Thus, it is not clear at the moment if the Red Queen may run faster or slower with the extra level of variation described here (Lively 2010).

## Supplementary information

S1 Methods

S2 Methods

S1 Table

S2 Table

S3 Table

S4 Table

S5 Table

Supplementary material titles

## Data Availability

Raw PacBio reads originating from the xD- and iD-locus haplotypes can be accessed under NCBI BioProject # PRJNA528268. Assembled haplotypes for the xD- and iD-locus can be found under GenBank accessions MK684166 and MK684167.
