# Supplementary material for: An alternative route of bacterial infection associated with a novel resistance locus in the Daphnia–Pasteuria host–parasite system
Source: Heredity (Edinb). 2020 Jun 19;125(4):173–83. doi: 10.1038/s41437-020-0332-x (PMC7490384; doi:10.1038/s41437-020-0332-x)
Supplement: Supplementary file 1 — S1 Methods [file 41437_2020_332_MOESM1_ESM.docx]

**S1 Methods** Fine Mapping

**Scoring of attachment phenotypes for fine mapping**

Resistance and susceptibility of *D. magna* individuals to *P. ramosa* P15 were assessed via the spore attachment test described in the Methods section.

However, due to the within clone variance observed in the spore attachment and because spores tend to stay longer in the hindgut even when not attached before being expelled, we tested attachment of individuals of 347 clones in the Recombinant Panel, independently, and scored the results with two distinct methods. The two assessments where done by two different observers, which were blinded to each other results.

Binary method – the observer classifies the tested *D. magna* individuals as either resistant (0) or susceptible (1). This is the method by which *P. ramosa* attachment to *D. magna* host foregut was defined in previous studies (e.g. Duneau *et al.*, 2011; Luijckx *et al.*, 2012; Bento *et al*. 2017) and was also the method used to define hindgut attachment in all other experiments described here.

Quantitative method – the observer evaluates the quality of attachment in the tested *D. magna* individuals and classifies the individual on a scale from 1 (no visible attachment) to 10 (full attachment throughout the hindgut).

We validated the quantitative method on of scoring attachment by testing the correlation between the mean of the binary scoring method and the median of the quantitative attachment scoring method. We used the 347 clones of the recombinant panel, which were tested with both methods. The results show that the binary and quantitative scoring methods are consistent with each other and that the correlation is strong (Pearson *r* = 0.73, n = 347; *p<*0.001).

Fine mapping

After five individuals of each *D. magna* clone were scored with the quantitative scoring method, clones were sorted by their median score and results were aligned to the mean of the results per clone from the binary method (S4 Table).

Clones that were fully resistant (i.e. mean of 0 with the binary method and median of 1with qualitative method) or fully susceptible (i.e. mean of 1.0 with the binary method and median of 10 with the qualitative method) were selected to proceed with the breakpoint mapping (S5 Table).

Out of 112 clones selected as fully resistant or fully susceptible there were 9 clones in which all genetic markers showed a genotype which was opposite of the observed phenotype (i.e. clone has resistant phenotype but all markers had genotype of susceptible parent or heterozygote, or clone has susceptible phenotype and all markers had genotype of resistant parent). These clones were classified as escapers and excluded from the breakpoint mapping analysis (S5 Table). Escapers are likely clones that have been mixed up during the 10-year history of this mapping panel.

The final interval of the D-locus therefore results from the breakpoint mapping of the remaining 104 Recombinant Panel clones (S5 Table).
