# Supplementary material for: An alternative route of bacterial infection associated with a novel resistance locus in the Daphnia–Pasteuria host–parasite system
Source: Heredity (Edinb). 2020 Jun 19;125(4):173–83. doi: 10.1038/s41437-020-0332-x (PMC7490384; doi:10.1038/s41437-020-0332-x)
Supplement: Supplementary file 8 — Supplementary material titles [file 41437_2020_332_MOESM8_ESM.docx]

**Supplementary Materials**

**S1 Methods –** Fine mapping methods

**S2 Methods** – Genomic characterization of the D-locus

**S1 Table –** Attachment of *Pasteuria ramosa* C1, C19 and P15 genotypes to the foregut and hindgut of 174 *Daphnia magna* genotypes from the “*Daphnia magna* Diversity Panel.” For each host clone, the consensus phenotype is given (S = attachment (= Susceptibility) and R = no attachment (= Resistance)), as is the number of replicates with positive attachment and the total number of replicates per host clone tested.

**S2 Table** – Results from P15-hindgut attachment and infection trials with *Pasteuria* P15 genotype in *Daphnia magna* females from the F2 recombinant panel.

**S3 Table –** Results of attachment tests for *Pasteuria ramosa* C19 and P15 in the F2 *Daphnia magna* clones of the QTL panel (core panel and extended panel)*.* Tests for P15 foregut were all negative in the F2 panel. The total number of replicates tested for P15 foregut and hindgut attachment sites is the same. The second sheet of the excel file gives the segregation ratios for the core and extended panels, as reported in the result section. These calculations assume that attachment is either positive (>= 50 % attachment) or negative (< 50 % attachment).

**S4 Table** – Results of the quantitative analysis of *P. ramosa* P15 spore attachment to the hindgut of *D. magna* F2 recombinant clones and comparison to binary scoring method

**S5 Table** – Results of the fine mapping of the D-locus
